# Supplementary material for: Late gene therapy limits the restoration of retinal function in a mouse model of retinitis pigmentosa
Source: Nat Commun. 2023 Dec 12;14:8256. doi: 10.1038/s41467-023-44063-8 (PMC10716155; doi:10.1038/s41467-023-44063-8)
Supplement: Supplementary file 1 — Supplementary Information [file 41467_2023_44063_MOESM1_ESM.pdf]

Supplementary Figures

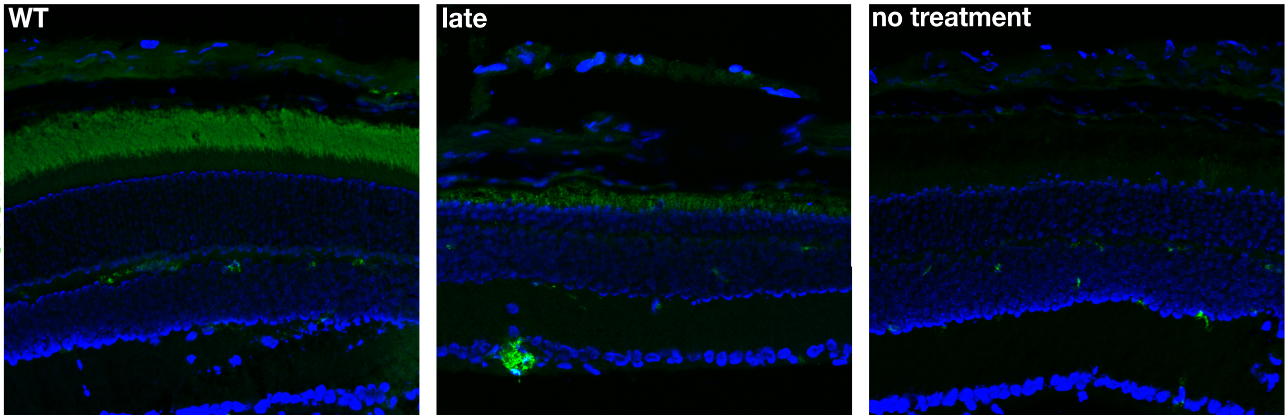

Figure S1:* Cnga1 *expression is restored with late treatment. *Confocal images of retinal cryosections from WT, late treated* Cngb1$^{neo/neo}$ *mice, and untreated* Cngb1$^{neo/neo}$ *mice.* Cnga1 *expression in green and DAPI nuclei in blue. Similar results were obtained at earlier treatment timepoints.*

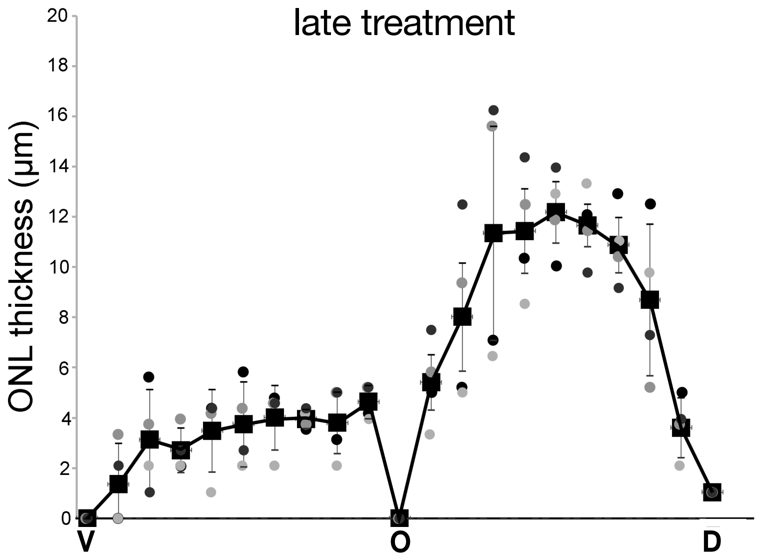

Figure S2: Following late treatment, ventral photoreceptors are less preserved than dorsal. *Quantification of outer nuclear layer thickness from cross sections spanning dorsal to ventral retina. Each square is the average between two measurements (circles) taken from two retinas (grey and black). Qualitatively similar results were obtained at earlier treatment timepoints. Source data are provided as a Source Data file.*

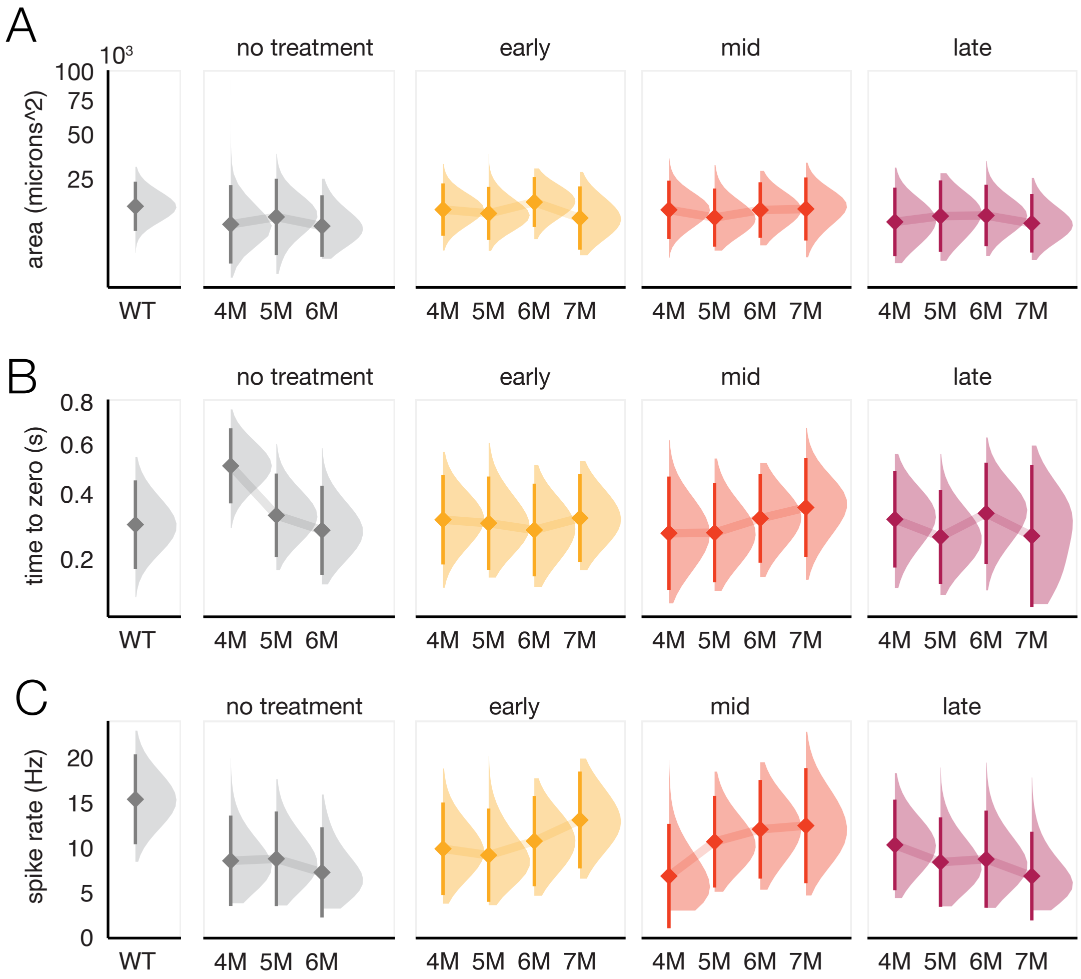

Figure S3: Mesopic receptive field measurements are stable following treatment, but response gain under mesopic conditions does not recover following late treatment. Similar to Figure 4, *the panels show distributions of A) time to zero, B) receptive field size, and C) gain, (diamond is the mean and bar is +/- SD) in WT, untreated, early, mid and late treated retinas from 4 to 7M. Light level was 100 Rh*/rod/s.*

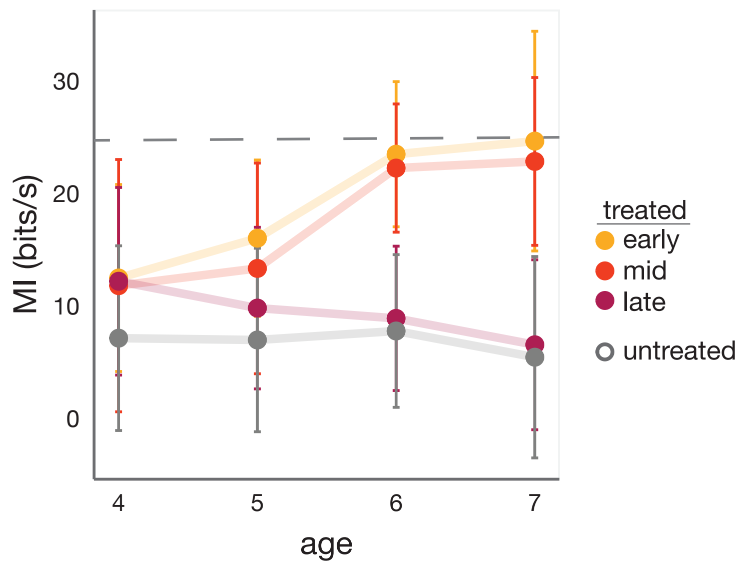

Figure S4: Consistent with results at photopic and scotopic light levels, late treatment fails to restore RGC information rates in response to mesopic repeating checkerboard stimuli. *Mean +/- 2 SD mutual information rate of RGC responses (n = 2-6 retinas) at mesopic light level (100 Rh*/rod/s) from the 10% most informative RGCs. Dashed line indicates the mean information rate observed in RGC responses from WT retinas.*

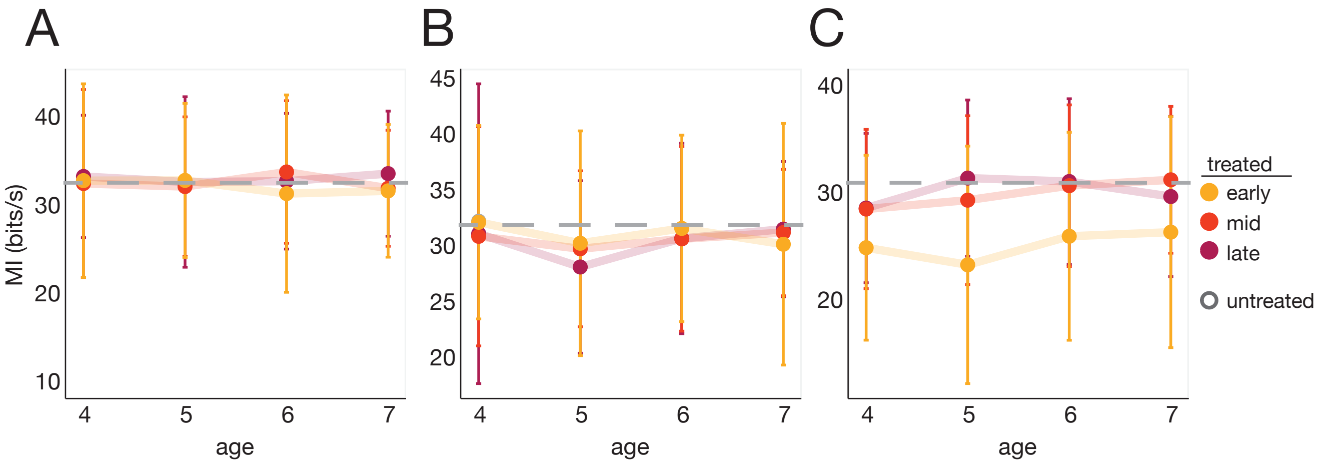

Figure S5: RGC information rates in response to natural movies are higher and more robust to degeneration than checkerboard noise. *Following previous work (Scalabrino et al., 2022), we computed the median information rate for the 10% most informative RGCs. In untreated* Cngb1$^{neo/neo}$ *retinas, this population of RGCs exhibited the clearest changes in information rates compared to control retinas (Scalabrino et al.,2022). Consistent with this previous study, we observed that information rates were higher for natural movies than for checkerboard noise and less sensitive to rod photoreceptor loss. Correspondingly, for early, mid, and late treatment time points, RGC information rates for natural movies matched those of WT at all ages tested under photopic and mesopic conditions (A-B). However, under scotopic conditions, there was a reduction in the information rates at every age tested for the late treatment, but not for the early and mid treatment time points (C). In each panel, the points indicate the mean +/- 2 SD of the information rate of RGC responses (n = 2-6 retinas) from the 10% most informative RGCs. Dashed line indicates the mean information rate observed in RGC responses from WT retinas.*
