# Supplementary material for: Late gene therapy limits the restoration of retinal function in a mouse model of retinitis pigmentosa
Source: Nat Commun. 2023 Dec 12;14:8256. doi: 10.1038/s41467-023-44063-8 (PMC10716155; doi:10.1038/s41467-023-44063-8)
Supplement: Supplementary file 3 — Reporting Summary [file 41467_2023_44063_MOESM3_ESM.pdf]

nature portfolio

Corresponding author(s): Greg Field

Last updated by author(s): Oct 25, 2023

Reporting Summary

Nature Portfolio wishes to improve the reproducibility of the work that we publish. This form provides structure for consistency and transparency in reporting. For further information on Nature Portfolio policies, see our Editorial Policies and the Editorial Policy Checklist.

Statistics

For all statistical analyses, confirm that the following items are present in the figure legend, table legend, main text, or Methods section.

n/a	Confirmed	
☐	☒	The exact sample size (n) for each experimental group/condition, given as a discrete number and unit of measurement
☐	☒	A statement on whether measurements were taken from distinct samples or whether the same sample was measured repeatedly
☐	☒	The statistical test(s) used AND whether they are one- or two-sided<br>*Only common tests should be described solely by name; describe more complex techniques in the Methods section.*
☐	☒	A description of all covariates tested
☐	☒	A description of any assumptions or corrections, such as tests of normality and adjustment for multiple comparisons
☐	☒	A full description of the statistical parameters including central tendency (e.g. means) or other basic estimates (e.g. regression coefficient) AND variation (e.g. standard deviation) or associated estimates of uncertainty (e.g. confidence intervals)
☐	☒	For null hypothesis testing, the test statistic (e.g. F, t, r) with confidence intervals, effect sizes, degrees of freedom and P value noted<br>*Give P values as exact values whenever suitable.*
☒	☐	For Bayesian analysis, information on the choice of priors and Markov chain Monte Carlo settings
☒	☐	For hierarchical and complex designs, identification of the appropriate level for tests and full reporting of outcomes
☒	☐	Estimates of effect sizes (e.g. Cohen's d, Pearson's r), indicating how they were calculated

Our web collection on statistics for biologists contains articles on many of the points above.

Software and code

Policy information about availability of computer code

Data collection	Visual stimuli were displayed using publicly available MGL codebase available at: https://gru.stanford.edu/doku.php/mgl/overview Multielectrode array data were acquired with customized Lab View software. The acquisition code will be made available upon reasonable request to the corresponding author.
Data analysis	Spike sorting was performed using a customized java software. Data analyses was performed using custom written codes in Matlab. Source code used to generate figures are available in the github repository https://github.com/mishek-thapa/treatment_paper. Code used to calculate mutual information can be found in the github repository https://github.com/mishek-thapa/cng-data.

For manuscripts utilizing custom algorithms or software that are central to the research but not yet described in published literature, software must be made available to editors and reviewers. We strongly encourage code deposition in a community repository (e.g. GitHub). See the Nature Portfolio guidelines for submitting code & software for further information.

Data

Policy information about availability of data

All manuscripts must include a data availability statement. This statement should provide the following information, where applicable:

- Accession codes, unique identifiers, or web links for publicly available datasets
- A description of any restrictions on data availability
- For clinical datasets or third party data, please ensure that the statement adheres to our policy

Source data used to generate figures are available in the github repository https://github.com/mishek-thapa/treatment_paper. Raw data are available upon request by contacting the corresponding author due to the size of the dataset >1TB. The Cngb1neo/neo mouse model is available upon request by contacting the corresponding author. Raw images for Figures 1-3 can be found at https://doi.org/10.5061/dryad.rv15dv4bv.

Research involving human participants, their data, or biological material

Policy information about studies with human participants or human data. See also policy information about sex, gender (identity/presentation), and sexual orientation and race, ethnicity and racism.

Reporting on sex and gender	N/A
Reporting on race, ethnicity, or other socially relevant groupings	N/A
Population characteristics	N/A
Recruitment	N/A
Ethics oversight	N/A

Note that full information on the approval of the study protocol must also be provided in the manuscript.

Field-specific reporting

Please select the one below that is the best fit for your research. If you are not sure, read the appropriate sections before making your selection.

☒ Life sciences ☐ Behavioural & social sciences ☐ Ecological, evolutionary & environmental sciences

For a reference copy of the document with all sections, see nature.com/documents/nr-reporting-summary-flat.pdf

Life sciences study design

All studies must disclose on these points even when the disclosure is negative.

Sample size	21 female, 19 male; Sample size was chosen to have at least 3 replicates per experimental time point/treatment.
Data exclusions	No data were excluded from analysis.
Replication	Physiological experiments were run across at least 3 animals for each condition. All replication attempts were successful.
Randomization	All retinas with receptive fields passing the quality control tests (Scalabrino, 2022) were included in this analysis with no sub-sampling, and thus there was no requirement for randomization. The mixed effects model used accounts for retina-to-retina variability by adding each experiment as a random effect. This procedure permitted making broad-level inferences about the RGC populations without dependence on experimental variability.
Blinding	Blinding was not possible for this study because all retinal ganglion cells were including based on quality control test (Scalabrino, 2022).

Reporting for specific materials, systems and methods

We require information from authors about some types of materials, experimental systems and methods used in many studies. Here, indicate whether each material, system or method listed is relevant to your study. If you are not sure if a list item applies to your research, read the appropriate section before selecting a response.

Materials & experimental systems

n/a	Involved in the study
☐	☒ Antibodies
☒	☐ Eukaryotic cell lines
☒	☐ Palaeontology and archaeology
☐	☒ Animals and other organisms
☒	☐ Clinical data
☒	☐ Dual use research of concern
☒	☐ Plants

Methods

n/a	Involved in the study
☒	☐ ChIP-seq
☒	☐ Flow cytometry
☒	☐ MRI-based neuroimaging

Antibodies

Antibodies used

mCar (1:500, Millipore AB15282, RRID: 652 AB_1163387), PCP2 (1:500, Santa Cruz sc-137064, RRID: AB_2158439), CtBP2 (1:1000, BD Biosciences Cat# 612044, RRID:AB_399431), GFAP (1:400, Sigma-Aldrich Cat# G3893, RRID:AB_477010), CNGA1 (1:50, generously provided by R. Molday), Alexa Fluor donkey anti mouse 647 (1:500, Thermo Fisher Scientific Cat# A-31571, RRID:AB_162542), and Alexa Fluor donkey anti rabbit 555 (Thermo Fisher Scientific Cat# A-31572, RRID:AB_162543)

Validation

Antibody choice was based on previous publications (i.e. Morgans et al., 2000; Schmitz et al., 2000; Lewis et al., 2003; Scalabrino et al., 2022; Wang et al., 2019). Validations were performed by the manufacturer using western blot analysis for mCar, PCP2, CtBP2, and GFAP. Additionally, these antibodies are available on the RRID portal, which includes usage and citation metrics. CNGA1 was validated in NJ Cook et al., 1989.

Animals and other research organisms

Policy information about studies involving animals; ARRIVE guidelines recommended for reporting animal research, and Sex and Gender in Research

Laboratory animals

Cngb1neo/neo mice were crossed with UBC-cre/ERT28 (JAX stock #007001, RRID: IMSR_JAX:007001). Mice were used in 1 month (P30) incriments starting at 1 month of age and ending at 7 months of age. Mice were housed in a facility kept at approximately 72°F and 30-70% humidity with a 12h light/dark cycle and fed chow ad libitum.

Wild animals

No wild animals were used in the study.

Reporting on sex

The sex of the animal and a neuron’s cell type was considered by including them as interaction terms with the treatment conditions. This step enabled determining whether treatment conditions were associated with information rates and receptive field sizes in a sex-independent fashion. The model indicated that conclusions about the impact of genetic rescue on RGC signaling were insensitive to sex, experimental variability, and cell type.

Field-collected samples

No field collected samples were used in the study.

Ethics oversight

Animal procedures were approved by the Duke University Institutional Animal Care and Use Committee guidelines (protocol A084-21-04) and in accordance with guidelines provided by the Association for Research in Vision and Ophthalmology.

Note that full information on the approval of the study protocol must also be provided in the manuscript.
